# Supplementary material for: Large university with high COVID-19 incidence is not associated with excess cases in non-student population
Source: Sci Rep. 2022 Feb 28;12:3313. doi: 10.1038/s41598-022-07155-x (PMC8885693; doi:10.1038/s41598-022-07155-x)
Supplement: Supplementary file 1 — Supplementary Information. [file 41598_2022_7155_MOESM1_ESM.docx]

**Title** Large university with high COVID-19 incidence is not associated with excess cases in non-student population

**Authors**: Nita Bharti, Brian Lambert, Cara Exten, Christina Faust, Matt Ferrari, Anthony Robinson

Table S1: Selected social characteristics from seven counties in Central Pennsylvania for population from The American Community Survey 5-year data 2019 [1].

| County | Population size (2019 ACS) | % of population 25 yrs or older with Bachelor’s degree or higher | Population = or > 65 yrs |
| --- | --- | --- | --- |
| Blair | 123157 | 21.3% ± 1.0 | \| 23728 ± 266 (~21.7%) \| \| --- \| |
| Centre (all) | 161960 | 45.5% ± 1.1 | \| 21572 ± 211 (~13.3%) \| \| --- \| |
| Clearfield | 79908 | 15.1% ± 0.8 | \| 15344 ± 190 (~19.2%) \| \| --- \| |
| Clinton | 38915 | 19.5% ± 1.5 | \| 6920 ± 127 (~17.8%) \| \| --- \| |
| Huntingdon | 45369 | 17.4% ± 1.1 | \| 8914 ± 188 (19.6%) \| \| --- \| |
| Mifflin | 46276 | 11.9% ± 1.2 | \| 9389 ± 156 (20.3%) \| \| --- \| |
| Union | 45111 | 26.0% ± 1.8 | \| 7462 ± 185 (16.5%) \| \| --- \| |
| Centre non-student | 131466 | 31.6% | 20860 (15.9%) |
| Centre student | 30494 | 11.0% | 712 (2.33%) |

Figure S1: Time series of weekly COVID-19 cases in students as reported from surveillance and on-demand University-provided testing for the Fall semester of 2020. Dashed grey vertical lines indicate the first two weeks of student return for the Fall semester, including the first 10 days of classes, and comprise 7.9% of all 5,052 COVID-19 cases seen in students during the Fall semester of 2020. The peak number of cases occurred during week 6 of the Fall semester.


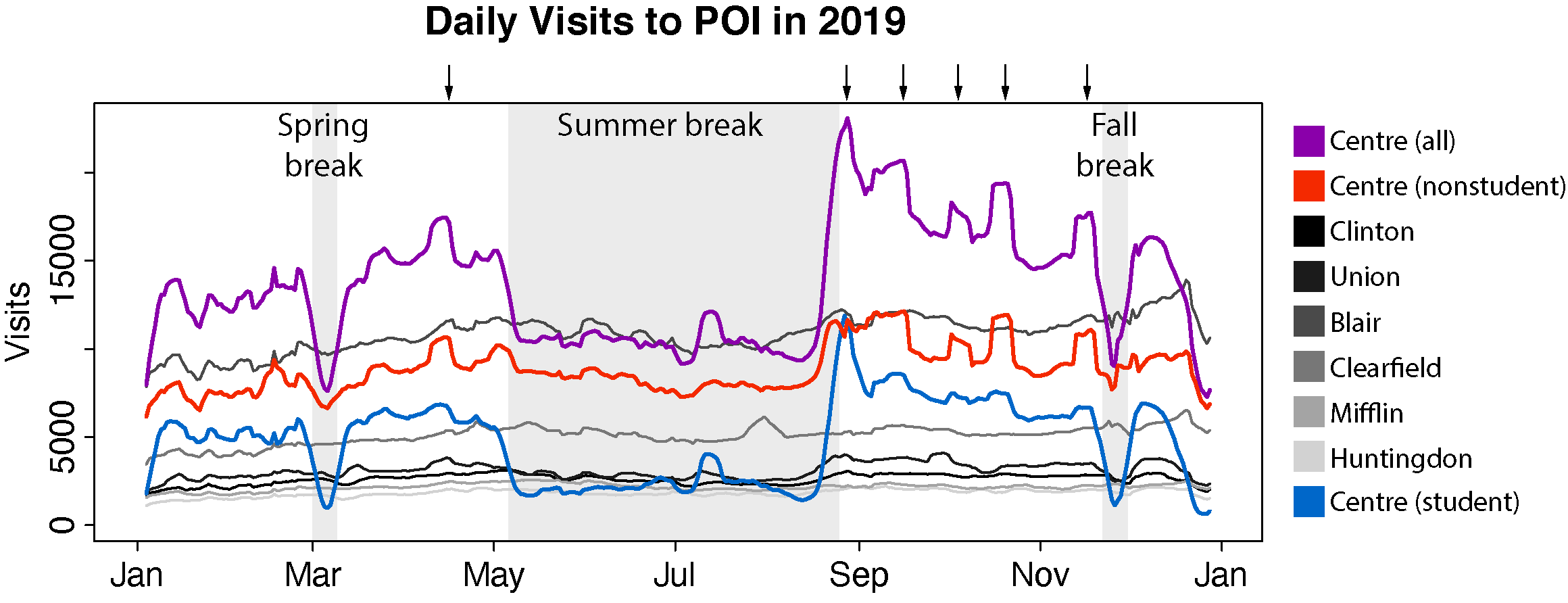


Figure S2: From SafeGraph [2], rolling seven day mean of total daily visits to points of interest per county and student- and non-student dominated CBGs within Centre County. Areas shaded in grey correspond to university breaks, arrows correspond to football games and a ticketed spring scrimmage.

References

1. **U.S. Census Bureau**. *American Community Survey 5-Year Data (2009-2019)*. 2020 Dec.

2. **SafeGraph**. *SafeGraph Data for Academics*. 2021.
